# Supplementary material for: Impact of Smoking Status on Mortality in STEMI Patients Undergoing Mechanical Reperfusion for STEMI: Insights from the ISACS–STEMI COVID-19 Registry
Source: J Clin Med. 2022 Nov 13;11(22):6722. doi: 10.3390/jcm11226722 (PMC9698021; doi:10.3390/jcm11226722)
Supplement: Supplementary file 1 [file jcm-11-06722-s001.zip › jcm-1879703-supplementary.pdf]

Supplementary Materials

Figure Legend:

Figure S1. Bar Graphs show the association between smoking status and postprocedural TIMI 3 flow in patients treated in 2019 (precovid era, left graph) and 2020 (covid era, right graph).
Figure S2. Bar Graphs show the association between smoking status and postprocedural TIMI 3 flow in young (left graph) and older (right graph) patients.
Figure S3. Bar Graphs show the association between smoking status and postprocedural TIMI 3 flow in in patients with low (left graph) and high (right graph) propensity score.
Figure S4. Bar Graphs show the association between smoking status and in-hospital mortality in patients treated in 2019 (precovid era, left graph) and 2020 (covid era, right graph).
Figure S5. Bar Graphs show the association between smoking status and in-hospital mortality in young (left graph) and older (right graph) patients.
Figure S6. Bar Graphs show the association between smoking status and in-hospital mortality in patients with low (right graph) and high (left graph) propensity score.
Figure S7. Kaplan-Meier survival curves according to smoking status in patients treated in 2019 (left graph) and 2020 (right graph).
Figure S8. Kaplan-Meier survival curves according to smoking status in young (left graph) and older (right graph) patients.
Figure S9. Kaplan-Meier survival curves according to smoking status in patients with low (left graph) and high (right graph) propensity score.

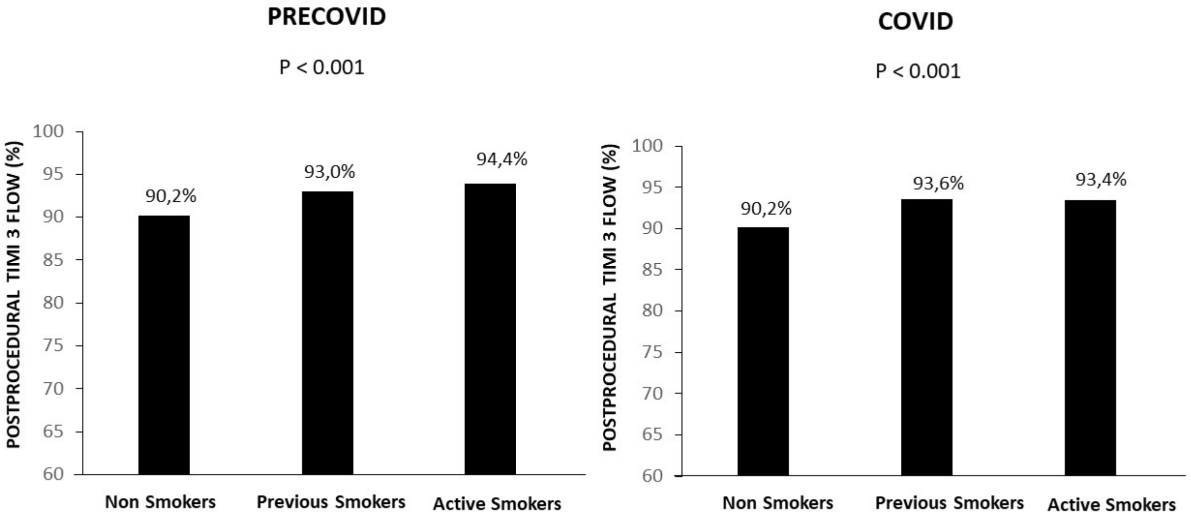

Figure S1. Bar Graphs show the association between smoking status and postprocedural TIMI 3 flow in patients treated in 2019 (precovid era, left graph) and 2020 (covid era, right graph).

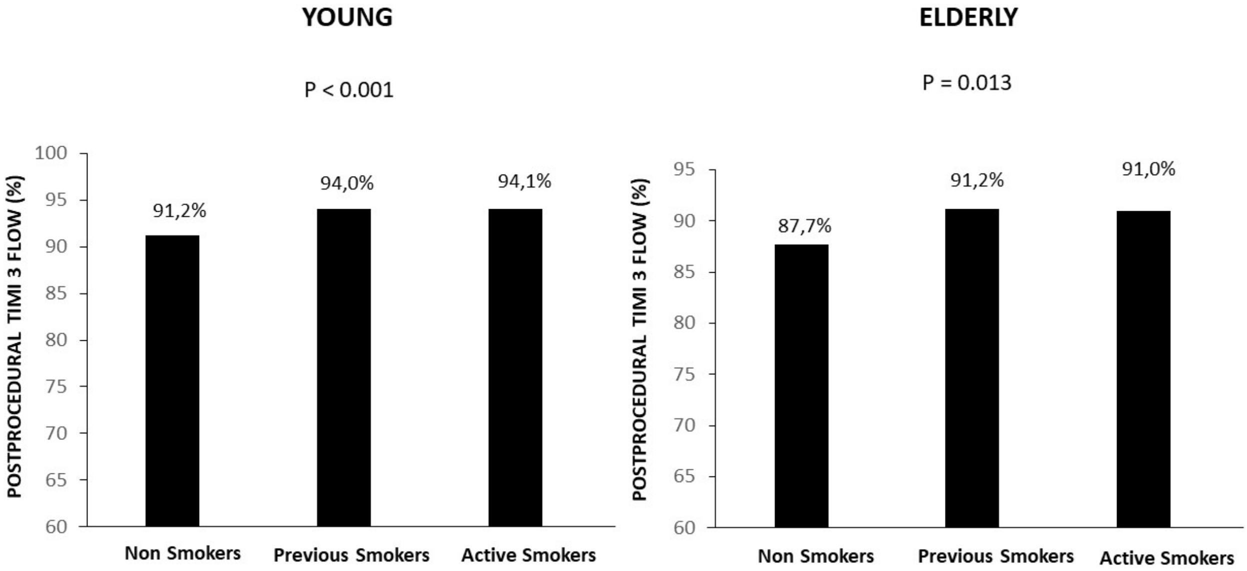

Figure S2. Bar Graphs show the association between smoking status and postprocedural TIMI 3 flow in young (left graph) and older (right graph) patients.

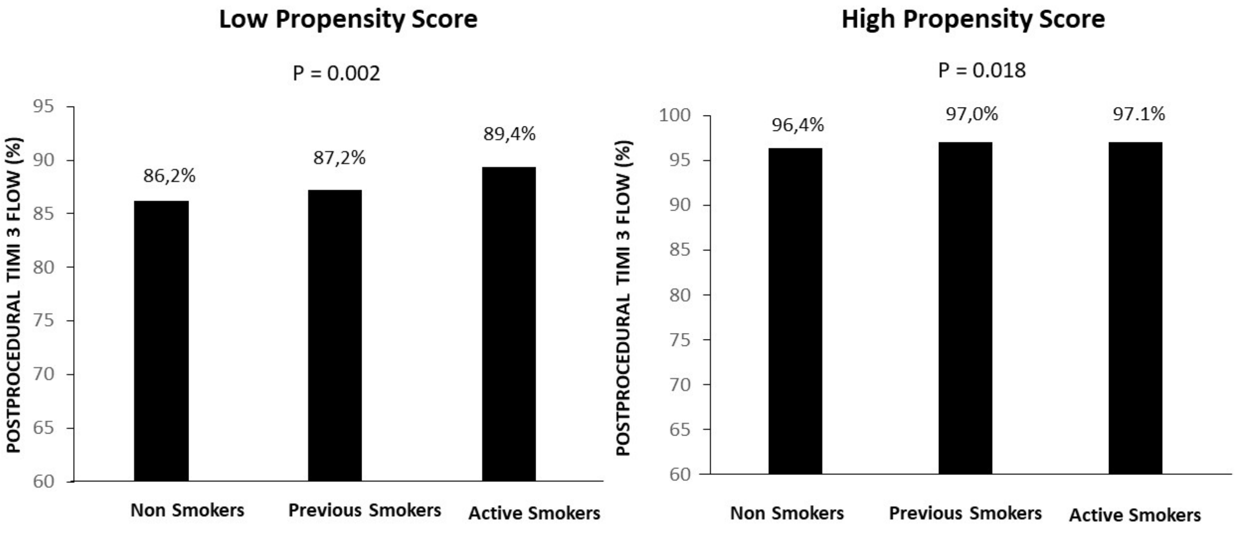

Figure S3. Bar Graphs show the association between smoking status and postprocedural TIMI 3 flow in in patients with low (left graph) and high (right graph) propensity score.

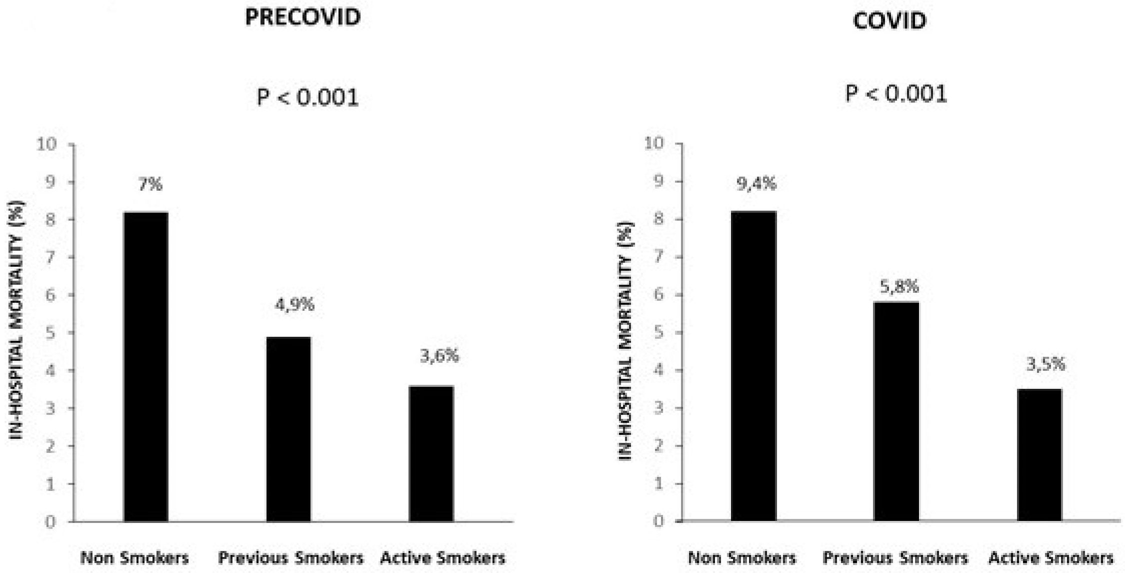

Figure S4. Bar Graphs show the association between smoking status and in-hospital mortality in patients treated in 2019 (precovid era, left graph) and 2020 (covid era, right graph).

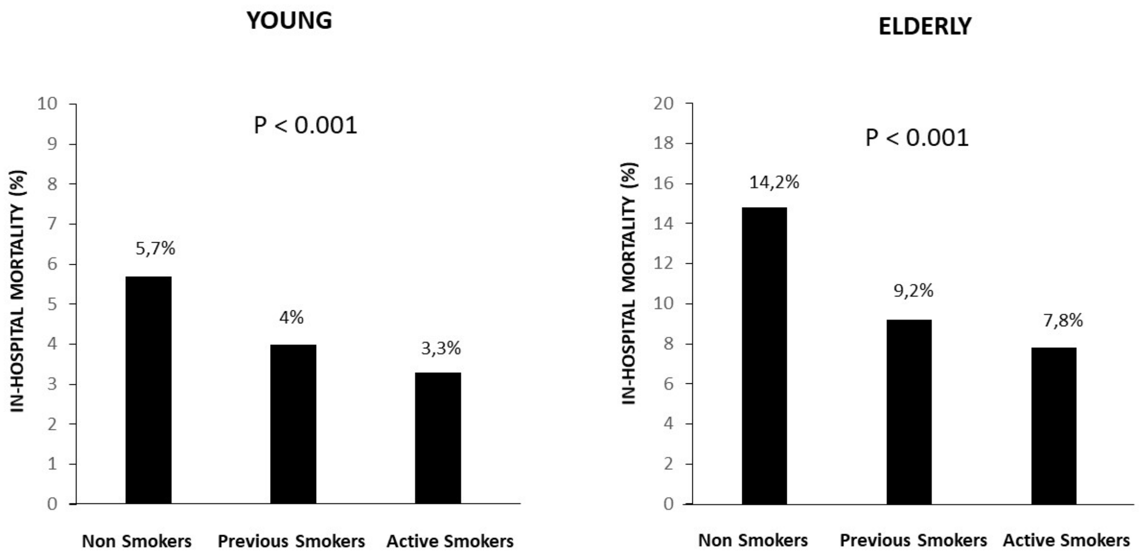

Figure S5. Bar Graphs show the association between smoking status and in-hospital mortality in young (left graph) and older (right graph) patients.

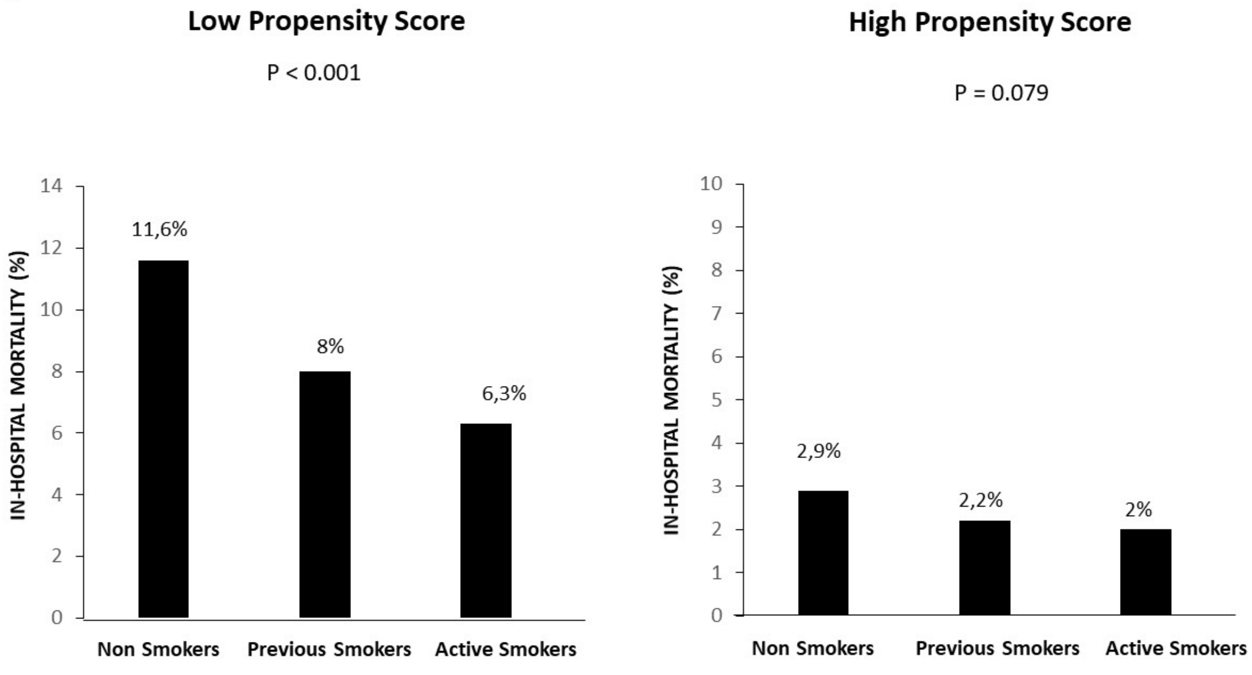

Figure S6. Bar Graphs show the association between smoking status and in-hospital mortality in patients with low (right graph) and high (left graph) propensity score.

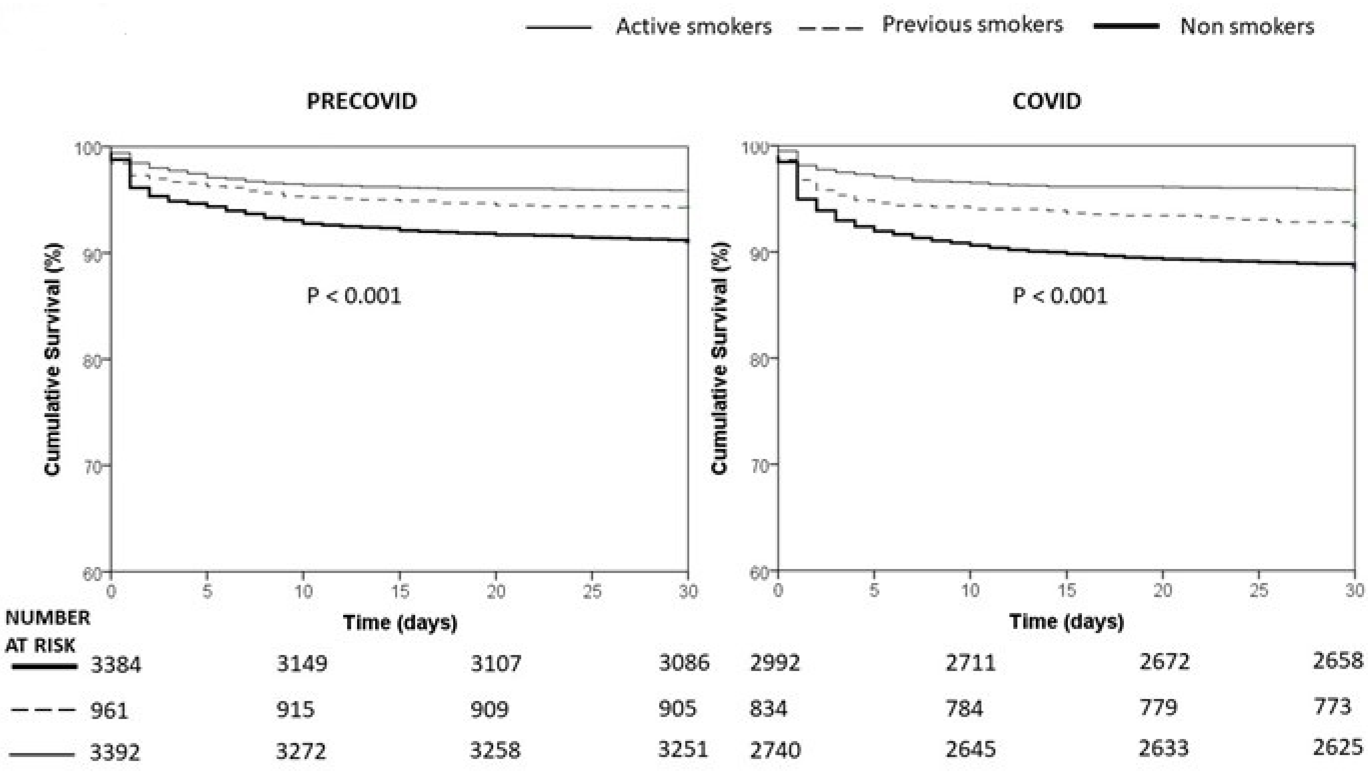

Figure S7. Kaplan-Meier survival curves according to smoking status in patients treated in 2019 (left graph) and 2020 (right graph).

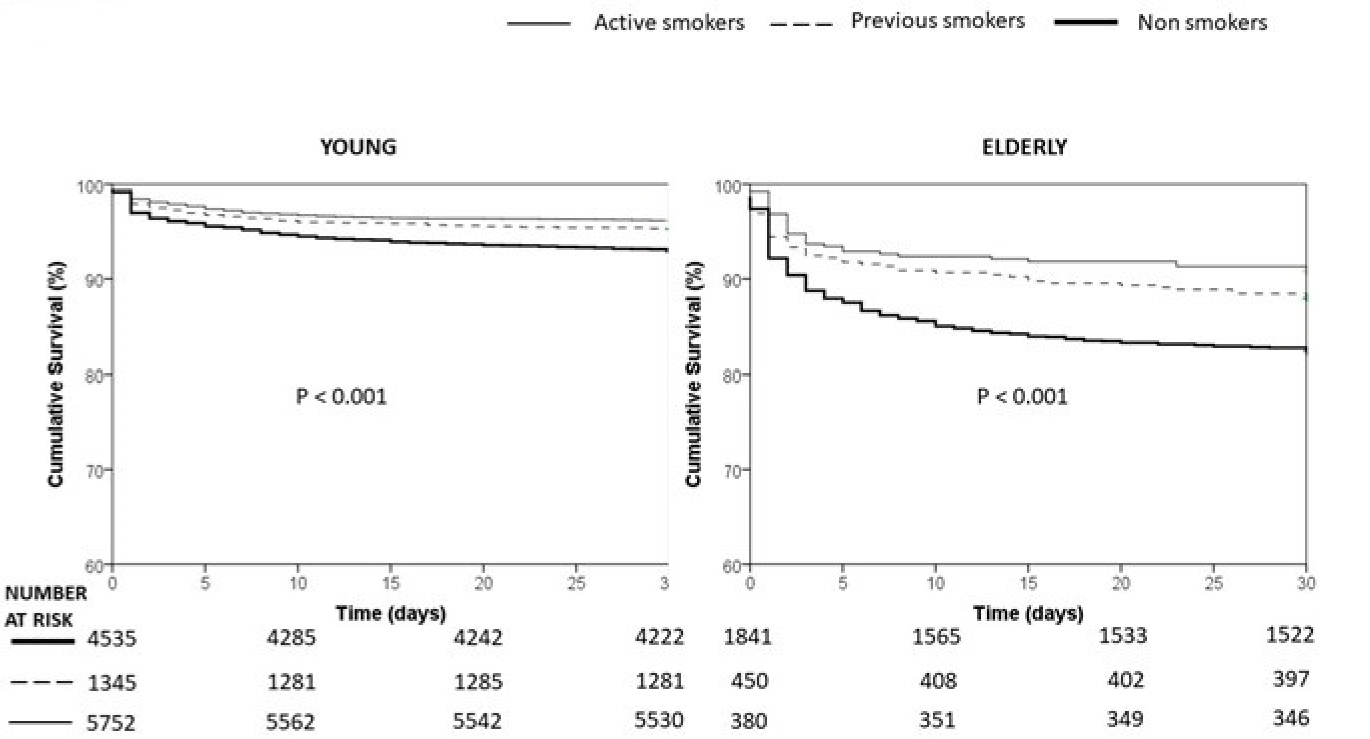

Figure S8. Kaplan-Meier survival curves according to smoking status in young (left graph) and older (right graph) patients.

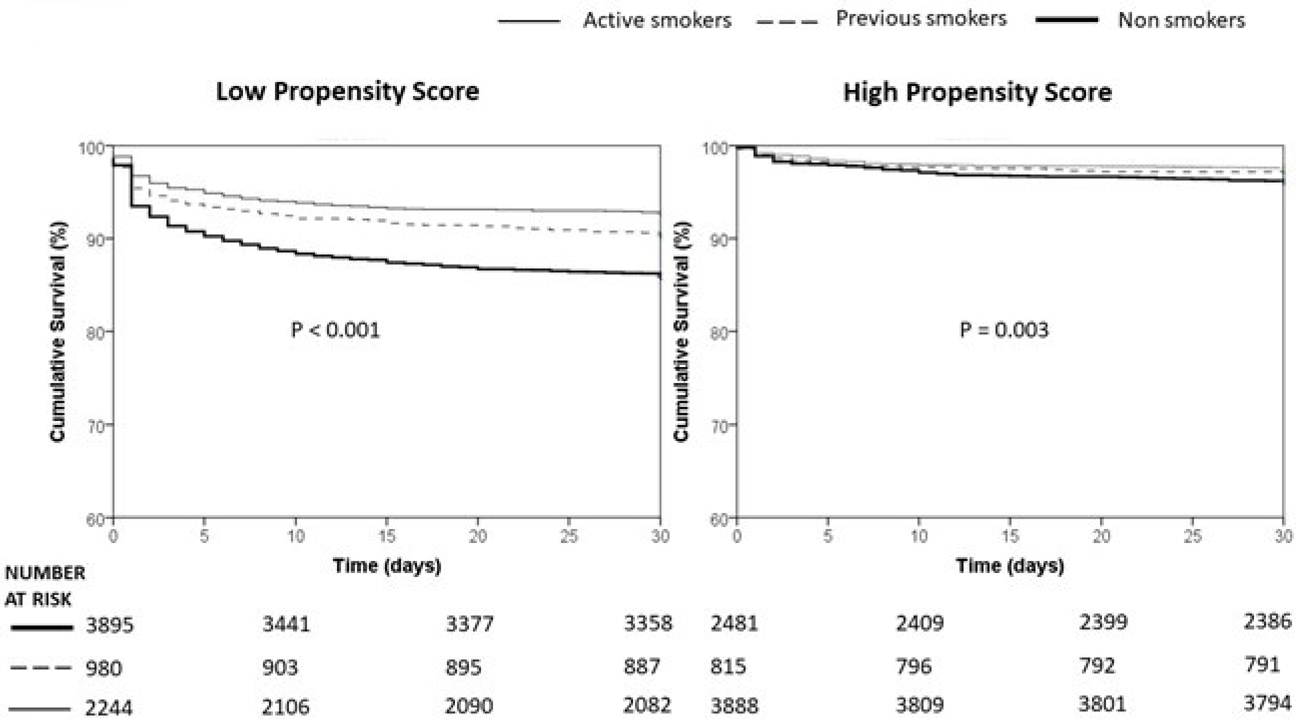

Figure S9. Kaplan-Meier survival curves according to smoking status in patients with low (left graph) and high (right graph) propensity score.
